# Nitrogen turnover and N_2_O/N_2_ ratio of three contrasting tropical soils amended with biochar

**DOI:** 10.1016/j.geoderma.2019.04.007

**Published:** 2019-08-15

**Authors:** Bernard Fungo, Zhe Chen, Klaus Butterbach-Bahl, Johannes Lehmannn, Gustavo Saiz, Víctor Braojos, Allison Kolar, Tatjana F. Rittl, Moses Tenywa, Karsten Kalbitz, Henry Neufeldt, Michael Dannenmann

**Affiliations:** a NationalAgricultural Research Organization (NARO), P. O. Box 1752, Kampala, Uganda; b Institute for Biodiversity and Ecosystem Dynamics (IBED), Faculty of Science, University of Amsterdam, Science Park 904, Amsterdam, the Netherlands; c World Agroforestry Center (ICRAF), P. O. Box 30677, 00100, United Nations Avenue, Gigiri, Nairobi, Kenya; d Institute for Meteorology and Climate Research, Atmospheric Environmental Research (IMK-IFU), Karlsruhe Institute of Technology (KIT), Kreuzeckbahnstrasse 19, Garmisch-Partenkirchen 82467, Germany; e Department of Crop and Soil Sciences, Cornell University, Bradfield Hall, Ithaca, NY 14853, USA; f Soil Resources and Land Use, Institute of Soil Science and Site Ecology, Dresden University of Technology, PiennerStrasse 19, 01737 Tharandt, Germany; g International Livestock Research Institute (ILRI), P.O. Box 30709, Nairobi 00100, Kenya; h Collage of Agricultural and Environmental Sciences (CAES), Makerere University, P. O. Box 7062, Kampala, Uganda; i Department of Soil Sciences, University of São Paulo, AvenidaPádua Dias, P.O. Box 9, Piracicaba, Brazil; j UNEP DTU Partnership, Copenhagen, Denmark; k Departamento de Química Ambiental, Universidad Católica de Concepción UCSC, Chile

**Keywords:** Nitrification, Ammonification, Denitrification, N_2_O protonation, ^15^N pool dilution method, Di‑nitrogen (N_2_)

## Abstract

Biochar has been reported to reduce emission of nitrous oxide (N_2_O) from soils, but the mechanisms responsible remain fragmentary. For example, it is unclear how biochar effects on N_2_O emissions are mediated through biochar effects on soil gross N turnover rates. Hence, we conducted an incubation study with three contrasting agricultural soils from Kenya (an Acrisol cultivated for 10-years (Acrisol10); an Acrisol cultivated for over 100-years (Acrisol100); a Ferralsol cultivated for over 100 years (Ferralsol)). The soils were amended with biochar at either 2% or 4% w/w. The ^15^N pool dilution technique was used to quantify gross N mineralization and nitrification and microbial consumption of extractable N over a 20-day incubation period at 25 °C and 70% water holding capacity of the soil, accompanied by N_2_O emissions measurements. Direct measurements of N_2_ emissions were conducted using the helium gas flow soil core method. N_2_O emissions varied across soils with higher emissions in Acrisols than in Ferralsols. Addition of 2% biochar reduced N_2_O emissions in all soils by 53 to 78% with no significant further reduction induced by addition at 4%. Biochar effects on soil nitrate concentrations were highly variable across soils, ranging from a reduction, no effect and an increase. Biochar addition stimulated gross N mineralization in Acrisol-10 and Acrisol-100 soils at both addition rates with no effect observed for the Ferralsol. In contrast, gross nitrification was stimulated in only one soil but only at a 4% application rate. Also, biochar effects on increased NH_4_^+^ immobilization and NO_3_^−^consumption strongly varied across the three investigated soils. The variable and bidirectional biochar effects on gross N turnover in conjunction with the unambiguous and consistent reduction of N_2_O emissions suggested that the inhibiting effect of biochar on soil N_2_O emission seemed to be decoupled from gross microbial N turnover processes. With biochar application, N_2_ emissions were about an order of magnitude higher for Acrisol-10 soils compared to Acrisol-100 and Ferralsol-100 soils. Our N_2_O and N_2_ flux data thus support an explanation of direct promotion of gross N_2_O reduction by biochar rather than effects on soil extractable N dynamics. Effects of biochar on soil extractable N and gross N turnover, however, might be highly variable across different soils as found here for three typical agricultural soils of Kenya.

## Highlights

• Biochar application consistently reduced N_2_O emissions from all soils.
• Biochar effects on gross N turnover were highly variable across soils.
• Biochar effects on N_2_O were decoupled from effects on mineral N availability.
• Kenyan Acrisols showed higher N_2_O emissions than Ferralsols.

## Graphical abstract

**Figure:**
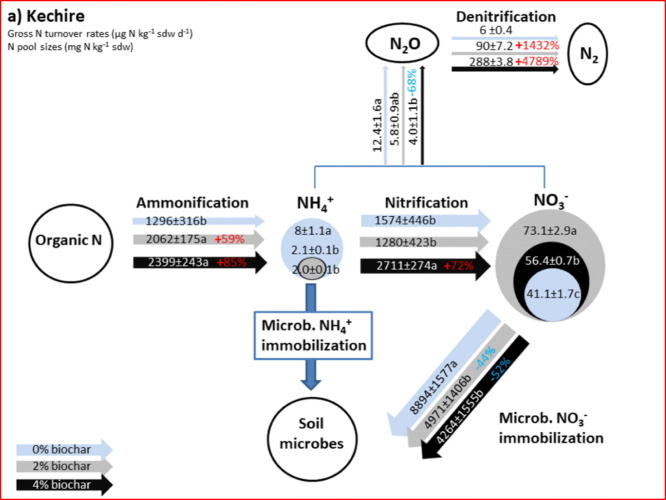

## 1 Introduction

Nitrous oxide (N_2_O) is a potent Long-Lived Greenhouse Gas (LLGHG), and involved in the destruction of stratospheric ozone (Ciais et al., 2013). Agricultural soils are an important source of atmospheric N_2_O, with denitrification representing the single most important biochemical process releasing N_2_O into the atmosphere (Butterbach-Bahl and Dannenmann, 2011; Harter et al., 2014a, Harter et al., 2014b). Measures for reducing N_2_O emission from agricultural soils such as biochar addition are increasingly considered to mitigate the impact of agriculture on climate change.

A number of factors affecting N_2_O emission in biochar-amended soils have been investigated, including feedstock, pyrolysis temperature, biochar pre-treatment, soil and biochar pH, soil type and soil moisture regime (Castaldi et al., 2011; Wu et al., 2012; Ameloot et al., 2013; Chen et al., 2017). For example, Yanai et al. (2007) suggested that a pH increase resulting from biochar addition could enhance N_2_O reductase activity, thereby increasing the reduction of N_2_O to N_2_in the last step of denitrification. Van Zwieten et al. (2009) hypothesized that metals present on biochar surfaces might act as catalysts in the reduction of N_2_O to N_2_. Physical adsorption of N_2_O and NO on activated coconut charcoal has also been reported (Bagreev et al., 2001; Hitoshi et al., 2002; Cornelissen et al., 2013). Case et al. (2015) found that the suppression of soil N_2_O emissions was not due to limitations of inorganic N availability in the soil caused by biochar-induced inorganic N immobilization. Furthermore, direct impacts of biochar on the activity of mineralizing and nitrifying microbes (Lehmann et al., 2011) may also occur but have, so far, hardly been investigated.

Using the ^15^N gas-flux method, Cayuela et al. (2013) observed a consistent reduction of the N_2_O/N_2_ ratio in 15 different soils after amendment with biochar, and proposed that biochar may act as an “*electron shuttle*”, facilitating the last step of denitrification (N_2_O to N_2_). According to Singh et al. (2010), sorption capacity of biochar through oxidative reactions on the biochar surfaces increase the effectiveness of biochar in reducing nitrate leaching, nitrification and N_2_O emissions. However, biochar effects on N_2_O emissions may also be mediated by its impact on prevailing soil conditions (Karhu et al., 2011; Yu et al., 2011; Case et al., 2012) that can influence the gross nitrogen turnover rates such as ammonification, nitrification, and inorganic N immobilization (Clough and Condron, 2010; Karhu et al., 2011). These conditions in turn exert feedbacks on N_2_O formation and consumption.

Knowledge on interactions between biochar addition, gross N turnover rates and soil N_2_O emissions is limited. Such detailed process-based understanding of N cycling in biochar-amended soils is important, since the ultimate effect of biochar addition on N gaseous losses could also depend on biochar's direct and/or indirect effect on ammonification, nitrification, microbial inorganic N immobilization, since these processes ultimately provide or remove substrate for denitrification and also impact N gas product ratios (Butterbach-Bahl and Dannenmann, 2011, Butterbach-Bahl et al., 2013). Furthermore, understanding biochar effects on gross N turnover is generally desirable to understand biochar effects on key soil functions such as fertility and nutrient retention (Clough and Condron, 2010). So far, the influences of biochar on gross N turnover rates and the N_2_O:N_2_ emission ratio, have only been considered separately in these earlier studies Cayuela et al., 2013, Case et al., 2015).

In this study, we provide data collected simultaneously on both the soil microbial gross N transformations as well as N_2_O and N_2_ emissions under the influence of biochar amendment and also measure the dynamics of all the soil mineral N pools. The objective of this study therefore was to provide a mechanistic understanding of biochar effects on the interplay of gross soil N mineralization, nitrification and immobilization as well as denitrification and the N_2_O:N_2_ product ratio. Three mineralogically contrasting tropical agricultural soils were used. We generally expected a coupling of soil gross N turnover (mainly gross nitrification) and N_2_O emissions, and that biochar impacts on gross N turnover would thus also affect N_2_O emissions. Specifically, we hypothesized that biochar addition to soil would (1) decrease nitrification and soil nitrate availability due to increased immobilization of mineral N; (2) decrease soil N_2_O emissions due to reduced total denitrification.

## 2 Materials and methods

### 2.1 Preparation of the biochar and soils

The feedstock from eucalyptus wood was chopped and ground into 5 mm-sized particles and fed into a 600 *l* batch pyrolysis unit using argon as a sweep gas at a flow rate of one liter per minute. The pyrolysis unit was programmed to run with a ramp temperature rate of 5 °C per min, reaching maximum temperature of 550 °C and a dwell time of 2 h at maximum temperature before cooling to room temperature.

Three soil types with contrasting characteristics were sampled (0–0.2 m topsoil) at the following sites in Western Kenya; (i) Gambogi (E34° 57′37″and N00°09′34″an Acrisol under cultivation for ~100 years mainly with maize-beans intercropping hereafter, Acrisol-100), (ii) Kechire (E35°0′00'and N0° 4′0″, an Acrisol after approximately 10 years of conversion from tropical high forest to maize cultivation, Acrisol-10), and (iii) Yala (a Ferralsol also under maize-beans cultivation >100 years, Ferralsol-100). The properties of the biochar and soil at each site are presented in Table 1. All the three soils are characterized by high content of 1:1 type clay presence of highly insoluble minerals such as quartz sand and sesquioxides, and low CEC. The organic matter content (Acrisol 10 > Acrisol 100 > Ferralsol 100Yala) and clay content (Kechire<Gambogi<Yala) were the major distinguishing features among the soils. In addition, the presence of iron and aluminum oxides as well as low amounts of available calcium and magnesium ions characterized the Ferralsol.

**Table 1:** Properties of biochar and soils from three soils in western Kenya, which were used in the incubation experiment.

Soil property	Units	Biochar		Soils	
			Acrisol-10*	Acrisol-100#	Ferralsol
pH		6.31	6.68	6.01	5.39
EC(S)	uS m^−1^	19.6	12.2	8.80	12.5
N	g kg^−1^	0.27	2.8	2.6	2.1
P	mg kg^−1^	135	2.77	2.30	20.3
K	mg kg^−1^	1490	263	223	550
Ca	mg kg^−1^	1920	2130	1950	2100
Mg	mg kg^−1^	150	413	312	226
Mn	mg kg^−1^	188	499	782	600
S	mg kg^−1^	36.5	7.25	14.0	10.4
Cu	mg kg^−1^	0.77	7.58	1.97	6.85
B	mg kg^−1^	1.07	1.25	0.33	0.68
Zn	mg kg^−1^	108	11.7	13.5	15.1
Na	mg kg^−1^	180	16.5	15.9	20.7
Fe	mg kg^−1^	164	123	67.2	192.3
Al	mg kg^−1^	559	888	939	895
C.E.C	meq/100 g	18.2	21.0	16.2	15.3
C:N ration		3218	9.7	9.4	10.5
SOC	g kg^−1^	869	27.2	24.3	19.0
Sand	%	nd	61.2	30.7	22
Silt	%	nd	18.3	47.5	43
Clay	%	nd	20.5	21.8	35

nd = Not determined.

*Acrisol-10: Soil type is an Acrisol that has been under cultivation for 10 years.

# Acrisol-100: Soil type is an Acrisol that has been under cultivation for 100 years.

### 2.2 Experimental setup

The experiment consisted of nine treatments that were derived from the three soils (Acrisol 10, Acrisol 100 and Ferralsol) and three biochar addition rates (0, 2% and 4%w/w). The pH of the biochar was adjusted to that of the soil using diluted HCl. The pH of the soil-biochar mixture was monitored and correlation between delta-pH (difference between original and final pH of the soil) was not correlated with N_2_O emission (Data not shown). Then, air-dry sieved soils (2 mm mesh) were re-wetted to 40% of water holding capacity (WHC) and incubated at 25 °C for seven days before the start of the experiment to stabilize microbial processes. After the stabilization period, each treatment was prepared by adding the appropriate biochar rate to the bulk soil and mixed thoroughly.

The incubation was performed in two experiments that were run independently but under identical incubation conditions; Experiment 1 was used for ^15^N isotope labeling as a basis for the application of the ^15^N pool dilution technique (as described in more detail by Dannenmann et al., 2010; Dannenmann et al., 2011) to quantify gross N turnover (nitrification, ammonification and NH_4_^+^/NO_3_^−^consumption/immobilization, three replicates for each treatment) and the associated N_2_O emissions (six replicates for each treatment). Experiment 2 was deployed using the helium flow soil core method (Butterbach-Bahl et al., 2002; Dannenmann et al., 2008) to simultaneously measure N_2_O and N_2_ in order to determine the N_2_O/N_2_ ratio in soils which did not receive ^15^N additions with two replicates for each treatment. Only two analytical replicates were possible due to limited capacities of the Helium soil core system and the long time needed for gas exchange. However, all N_2_ flux measurements were average fluxes from seven simultaneously incubated soil cores so that spatial replication was comparably good (see below).

### 2.3 Gross rates of nitrogen turn-over and N_2_O production

Gross rates of ammonification, nitrification and inorganic N consumption were determined using the^15^N pool dilution technique as described in detail by Dannenmann et al. (2010). Briefly, 200 g samples of air-dry soil were placed in 500 cm^3^ incubation bottles fitted with rubber caps to allow for air tightness during gas sampling. The bottles were prepared in duplicates to allow for separate enrichment with either ^15^NO_3_^−^ or ^15^NH_4_^+^. After mixing the wet soil with biochar, the moisture content of the treatments was raised to 70% WHC w/w and maintained at that level throughout the experiment by daily weighing and replacing water lost by evaporation. The incubation bottles were placed in the thermostatically–controlled incubator maintained at 25 °C (the average daily soil temperature in western Kenya) throughout the 20-day experimental period.

Before destructive soil sampling, the incubation bottles were closed, gas-tight, using the rubber caps, and 10 ml of gas was sampled at 0, 30, 60 and 90 min after closing. The range of *R*^*2*^ values ranged from 0.75 to 0.99%. However, a flux was included in the analysis only if the *R*^*2*^ was >85%. The gas samples were collected using a 20-ml syringe and injected into pre-evacuated 10-ml gas vials. Analyses of gas samples were done using a gas chromatograph equipped with an Electron Capture Detector (ECD) for N_2_O analysis as described in detail by Yao et al. (2010). Nitrous oxide flux was calculated from linear changes of N_2_O concentrations in the headspace (Yao et al., 2010).

Immediately after gas sampling, the soils in the bottles were enriched with a solution of either K^15^NO_3_or (^15^NH_4_)_2_SO_4_ at 50 atom% enrichment. The Isotopically labelled solution was applied by spraying it onto the soil, accompanied by intensive mixing (Dannenmann et al., 2010). For each sample, half of the soil was extracted 1 h after enrichment (T_0_) and the second half was stored in an incubator at 25^0^C for 24 h before the second extraction (T_1_). Sixty grams of T_0_ and T_1_ samples were extracted with 120 ml of 0.5 M potassium sulphate (K_2_SO_4_) solution by end-to-end shaking for 60 min. All extracts were filtered through 0.45 μm syringe filters.

The diffusion method was used for subsequent trapping NH_4_^+^ or NO_3_^−^ as NH_3_ on acid traps made of ashless filter paper (Brooks et al. 1989; Dannenmann et al., 2006, Dannenmann et al., 2010). The ^14/15^N-ratio of the N captured on the dried filter papers was analyzed using an elemental analyzer coupled to a mass spectrometer as described in detail by Guo et al. (2013). Ammonium and nitrate concentrations in extracts were quantified using colorimetric auto-analysis (AQUAfast COD165 Thermoreactor, Thermofisher Scientific, USA) according to the VDLUFA method C 221 (Hoffmann, 1991). Gross rates of ammonification and nitrification were calculated using the equations given by Kirkham and Bartholomew (1954) using T_0_ and T_1_ data on N pools and ^15^N enrichment of ammonium and nitrate, respectively.

Furthermore, we calculated gross inorganic NH_4_^+^ and NO_3_^−^ consumption and then estimated immobilization of NH_4_^+^ by subtracting nitrification rates from NH_4_^+^ consumption rates (Davidson, 1992). We do not declare nitrate consumption to resemble biotic and/or abiotic nitrate consumption because other nitrate fates such as denitrification may be substantial in our incubations. Soil pH was determined using a pH meter after shaking a 1:2.5 w/v soil-to-water mixture and allowing it to stand overnight before measurement. Gas flux analyses and determination of gross N turnover were conducted immediately after biochar addition, and after 3, 7, and 20 days in triplicate. Overall, 216 jars with soil were used for this purpose in this experiment.

### 2.4 N_2_ emissions

Emission rates of N_2_ were determined by use of the helium gas flow soil core method (Butterbach-Bahl et al., 2002) with the modified setup for smaller soil samples and better representation of spatial variability described by Dannenmann et al. (2010). The method is based on the exchange of the soil and headspace atmospheres by a helium‑oxygen atmosphere containing only 25 ppm N_2_in an extremely gas-tight incubation system and the subsequent simultaneous automated detection of N_2_ concentration changes in the headspace above the cores by use of a pulse discharge helium ionization detector (PDHID) for N_2_.

The general set-up of the system consists of the steering unit, two vessels containing seven soil cores equipped for automated flushing both through the soil cores and headspace, automated sampling and the detection devices and systems. Details of the system and of the conditions for N_2_ analysis are described by Dannenmann et al. (2011). The soils were pre-treated as described above and placed in the seven cores (0.01 m^3^ volume each) of a single incubation vessel (soil moisture 70% WHC w/w incubation temperature: 25 °C).

After closing the vessels, the soil cores were flushed for 72 h to quantitatively remove N_2_ from the soil and headspace atmospheres. Subsequently, an artificial headspace atmosphere was created (5 h of flushing with 80% He, 20% O_2_, 25 ppm N_2_, 400 ppb N_2_O) and finally the concentration change of N_2_ in the two cuvettes was monitored automatically for 8 h on an hourly basis according to Butterbach-Bahl et al. (2002). Every sample gas analysis was accompanied by six automated calibration gas measurements of the gas chromatographs. For each treatment, two replicates (each consisting of combined N gas measurements from seven soil cores) were used. Before starting the measurement, the air-tightness of the system was checked with a parallel set-up containing empty vessels and soil core dummies made of steel; the inherent leakage rate of N_2_ was <20 μg N_2_-N m^−2^ h^−1^.

### 2.5 Data analysis

Calculation of cumulative fluxes during the incubation period was based on linear interpolation between measurements. All biogeochemical N data were expressed on a soil dry weight (sdw) basis. The main effect of biochar presence, biochar rate or soil type was tested using factorial ANOVA after natural log transformation, and individual means were separated by the methods of Least Significant Difference (LSD) at 95% level of confidence. Correlation analysis was used to assess the relationships between soil properties and N transformation processes and gaseous N products.

## 3 Results

### 3.1 Biochar and N_2_O emission

**Fig. 1:**
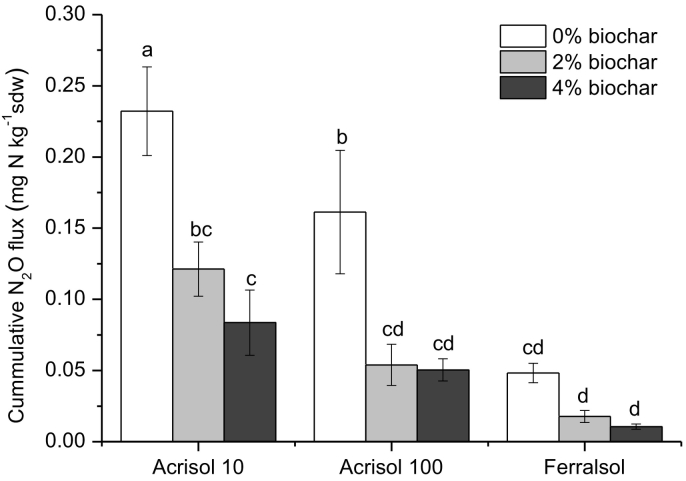
Cumulative N_2_O fluxes after 20-day incubation from three contrasting tropical agricultural soils after amendment with different quantities of biochar (w/w). Error bars are standard errors of the mean (n = 6). Different indices indicate significant differences between biochar addition treatments (*P* < 0.05, LSD test).

The cumulative N_2_O losses over the incubation period followed the order Acrisol10 > Acrisol-100 > Ferralsol (Fig. 1), i.e., decreased with decreasing soil organic carbon content (Table 1). The application of biochar reduced cumulative N_2_O emission (Fig. 1) by 53 to 78% across soils and biochar addition treatments. Increasing the application rate of biochar from 2% to 4%, however, did not significantly reduce cumulative N_2_O emissions from any of the three soils (Fig. 1). No significant correlations were found between various mineral concentrations in biochar and N_2_O emissions (Supplementation Material).

### 3.2 Extractable NO_3_^−^N and NH_4_^+^-N

**Fig. 2:**
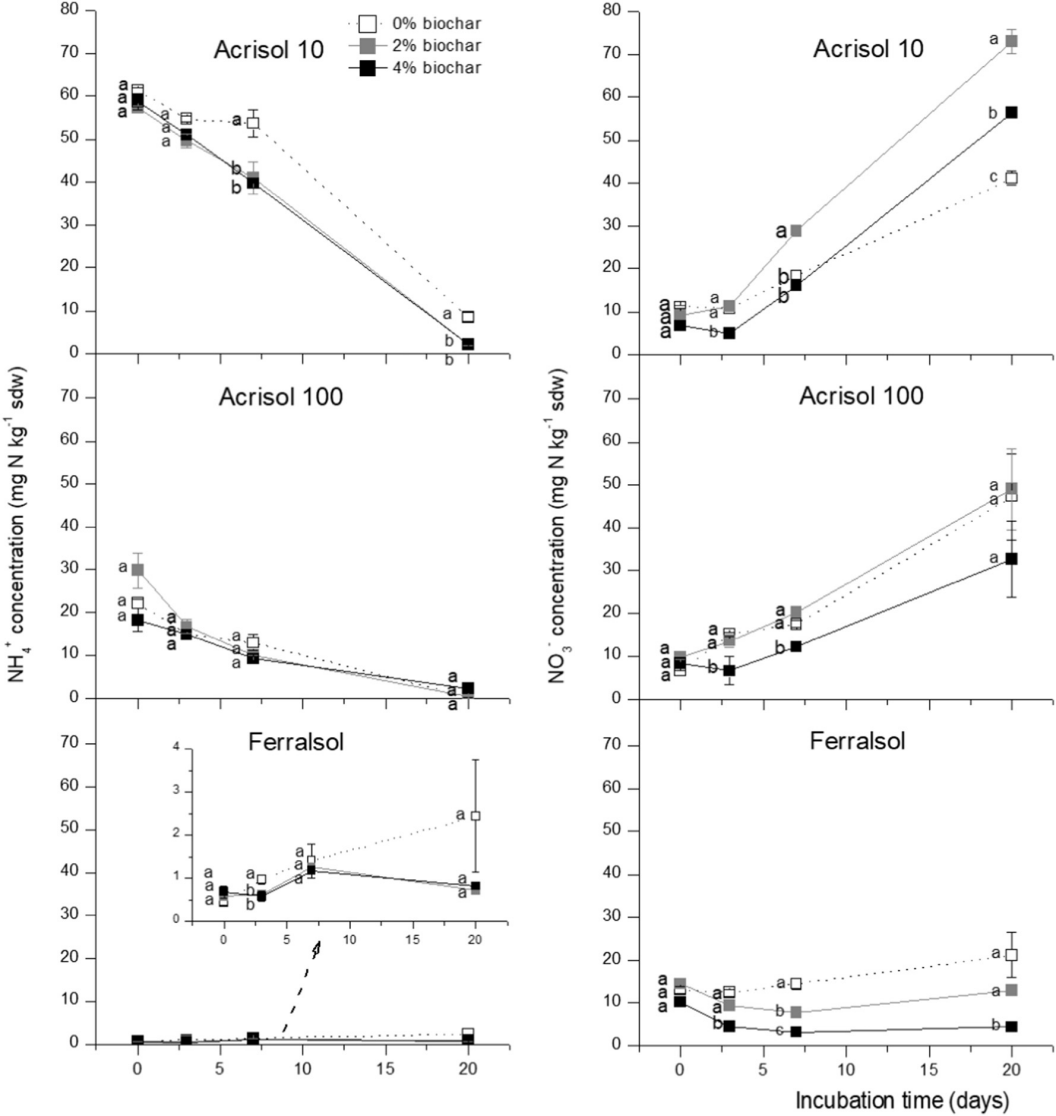
Concentrations of NO_3_^−^-N (A) and NH_4_^+^-N (B) during a 20-day incubation in three soils amended with 2% and 4%w/w biochar. Error bars represent standard error, and n = 6.

Fig. 2 illustrates the dynamics of soil NO_3_^−^-N and NH_4_^+^-N concentrations during the 20-day incubation period. All three soils showed comparable initial NO_3_^−^ concentrations of ca 10 mg N kg^−1^sdw, while initial NH_4_^+^-N concentrations strongly differed across soils with the pattern Acrisol10 > Acrisol100 > Ferralsol, with the latter showing extremely low NH_4_^+^ concentrations. For Acrisol10and Acrisol100 soils, NH_4_^+^ concentrations decreased throughout the incubation, while there was a parallel increase in NO_3_^−^concentrations in the same order of magnitude (Fig. 2). In contrast, the Ferralsol showed no pronounced change in soil mineral N concentrations. Towards the end of the incubation, biochar addition had resulted in increased NO_3_^−^ concentrations in Acrisol 10 but decreased NO_3_^−^ concentrations in the Acrisol100 (only for the high addition rate) and in the Ferralsol (for both addition rates) (Fig. 2). In contrast, soil NH_4_^+^concentrations were significantly reduced by biochar addition, but only for the Acrisol10 soil.

### 3.3 Gross ammonification and nitrification rates

**Fig. 3:**
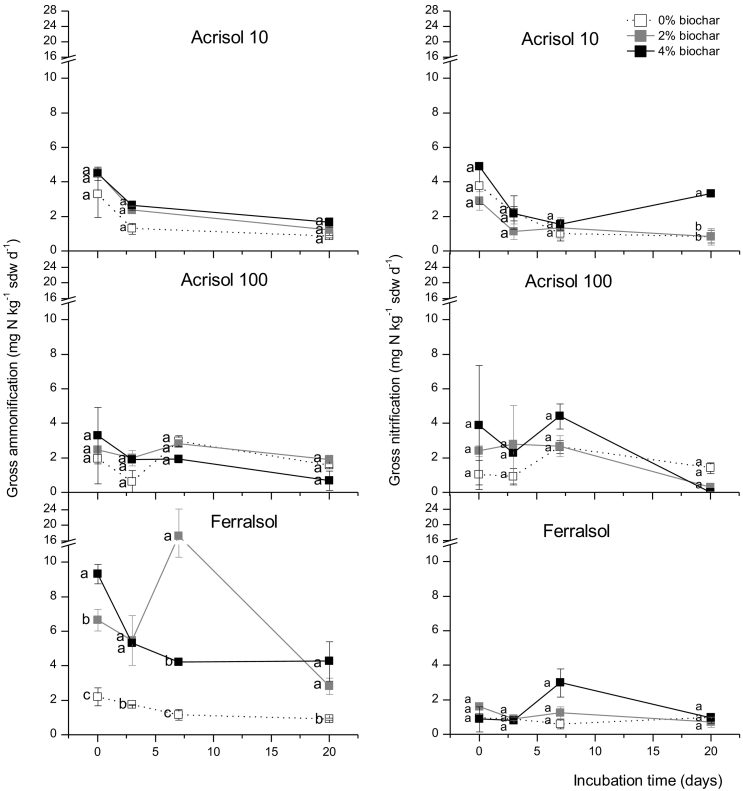
Gross ammonification (left panels) and nitrification (right panels) rates during a 20-day incubation in three soils amended with 2% and 4%w/w biochar. Error bars represent standard error, and n = 6.

For the Acrisol10 and Acrisol100 soils, gross nitrification rates were similar to gross ammonification rates, indicating a nitrate-oriented N cycle. In contrast, the Ferralsol showed gross nitrification rate to be significantly lower than gross ammonification (Fig. 3; Table 2). Overall, a biochar addition rate of 2% increased ammonification rates of Acrisol-10 (69%) and Ferralsol (639%) soils, with a similar effect of the high application rate of 4% (85% increase for Acrisol10 and 282% increase for Ferralsol) over the entire incubation period, while no persistent or unidirectional effect was observed for Acrisol100 (Fig. 3, Fig. 5).

**Table 2:** Cumulative nitrogen transformation over the 20-day incubation in three contrasting soils after amendment with different quantities of biochar (mg N kg^−1^sdw 20 days^−1^) with standard error in brackets. Cumulative N_2_O is given in the same unit.

N process	Soil type	0% biochar	2% biochar	4% biochar
Ammonification	Acrisol-10	27(7)^b^	43(4)^a^	50(5)^a^
	Acrisol 100	42(12)^a^	49(7)^a^	28(7)^b^
	Feralsol	27(7)^b^	199(101)^a^	103(11)^a^
Nitrification	Acrisol-10	33(9)^b^	27(9)^b^	57(6)^a^
	Acrisol 100	34(12)^a^	37(19)^a^	44(7)^a^
	Feralsol	17(3)^a^	22(7)^a^	36(14)a
NH_4_^+^-N immobilization	Acrisol-10	n.a.	n.a.	n.a.
	Acrisol 100	15(15)^b^	63(17)^a^	23(26)ab
	Feralsol	39(21)^b^	243(90)^a^	146(16)a
NO_3_^−^-N consumption	Acrisol-10	187(33)^a^	104(30)^b^	90(33)b
	Acrisol 100	49(48)^a^	151(99)^a^	38(27)a
	Feralsol	60(27)^a^	27(2)^a^	32(20)a
N_2_O fluxes	Acrisol-10	0.26(0.034)^a^	0.12(0.019)^ab^	0.08(0.023)^b^
	Acrisol 100	0.16(0.043)^a^	0.05(0.015)^b^	0.05(0.008)^b^
	Feralsol	0.05(0.007)^a^	0.02(0.004)^ab^	0.01(0.002)^b^

Values with similar superscripts are not significantly different.

With regard to gross nitrification rates, no persistent effects of biochar addition were generally observed over the incubation period (Fig. 3). Despite these variable effects, cumulative gross nitrification rates as calculated over the entire incubation period were significantly increased for all three soils at 4% biochar addition but not at 2% biochar addition (Table 2, Fig. 5).

### 3.4 Gross NH_4_^+^-N and NO_3_^—^N consumption rate

**Fig. 4:**
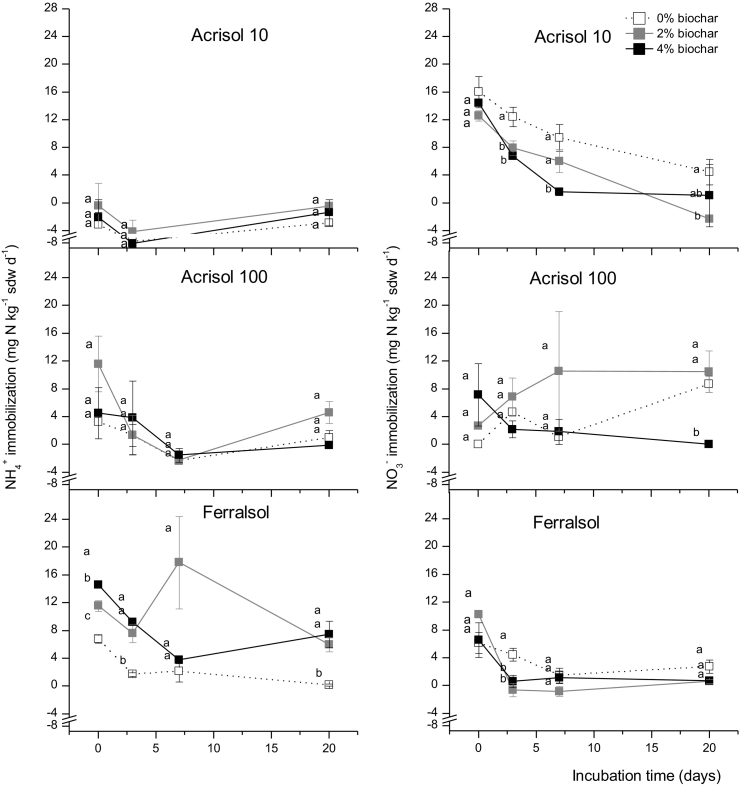
Immobilization of NH_4_^+^-N (A) and NO_3_^−^-N (B) during a 20-day incubation of three soils amended with 0%,2% and 4% w/w biochar. Error bars represent standard error of the mean (n = 6).

**Fig. 5:**
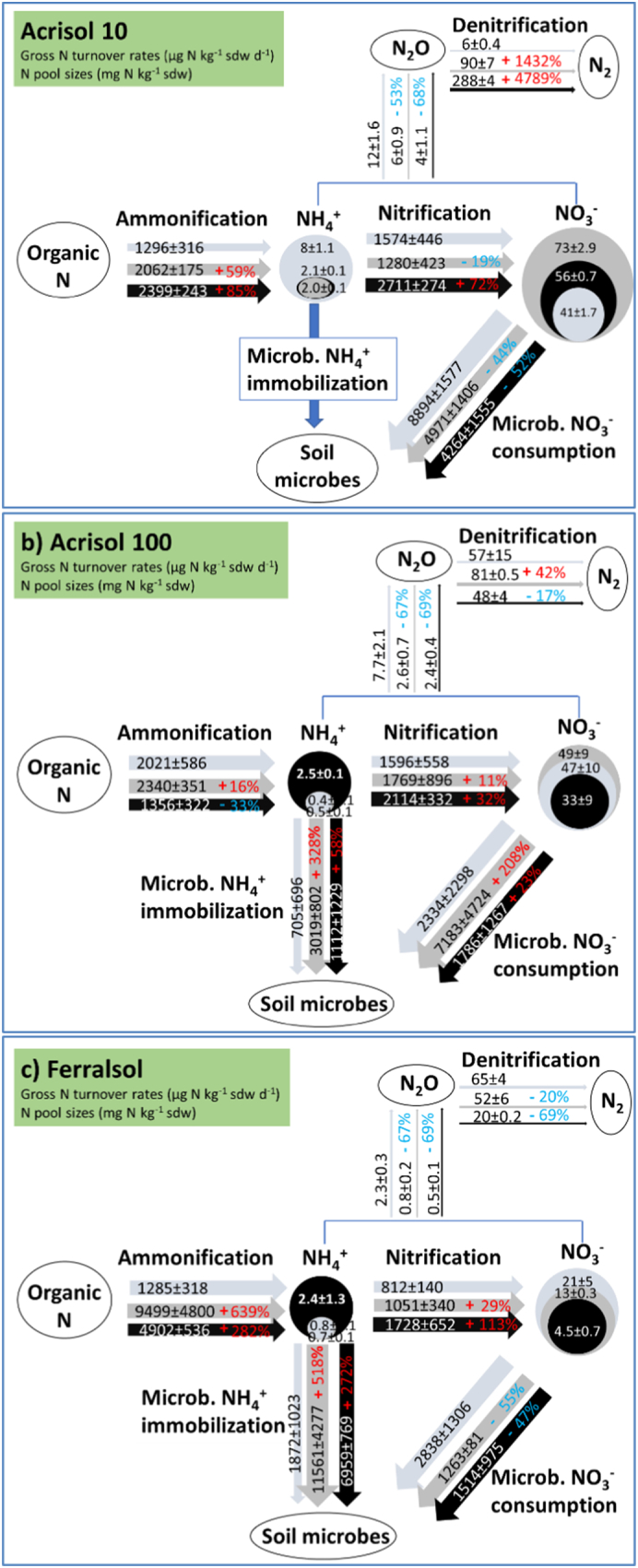
Mean gross N turnover rates (μg N kg^−1^sdwd^−1^) and N pool sizes (mg N kg^−1^sdw) for the three soils and three biochar treatments. Blue: 0% biochar addition (control treatment); Grey: 2% w/w biochar addition; Black: 4% w/w biochar addition. Thickness of process arrows and N pool signatures is representative for respective turnover rates and pool sizes. Biochar effects are provided as %change in red color (increase) or blue color (decrease). (For interpretation of the references to color in this figure legend, the reader is referred to the web version of this article.)

Ammonium immobilization rates as calculated from ammonium consumption minus gross nitrification resulted in significantly negative for the Acrisol 10, indicating N dynamics such as heterotrophic nitrification, i.e., a direct oxidation of organic N to NO_3_^−^ (Fig. 4). Hence, we did not calculate overall mean NH_4_^+^ immobilization fluxes for this soil (Fig. 5). Application of biochar increased NH_4_^+^-N immobilization only for the Ferralsol. In contrast, biochar generally increased NO_3_^−^ consumption for the Acrisol 100, decreased NO_3_^−^ consumption for the Acrisol100 and had no effect on NO_3_^−^ consumption in the Ferralsol (Table 2, Fig. 4, Fig. 5).

### 3.5 Dinitrogen losses

**Fig. 6:**
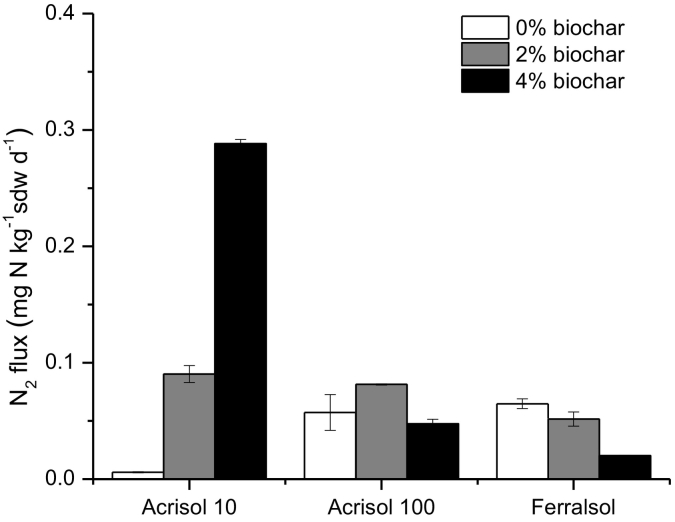
Dinitrogen (N_2_) emission rates from the three investigated soils at day 3 of the incubation period as influenced by biochar addition. (n = 4). Error bars are standard errors.

For dinitrogen losses, only two measurements are available so that from a statistical perspective we were unable to distinguish across soils and treatments. Dinitrogen emissions generally exceeded N_2_O emissions by at least an order of magnitude, so that they may represent total denitrification rates very well. Without biochar application, N_2_ emissions were about an order of magnitude lower for Acrisol10 compared to Acrisol100 and Ferralsol (Fig. 5, Fig. 6). Similar to other results on N turnover, biochar tended to exert variable effects on soil N_2_ emissions (Fig. 5). For the Acrisol-10, a very large increase in N_2_emissions was observed with increasing biochar addition (Fig. 5, Fig. 6). In contrast, for the Acrisol100, biochar addition did not change N_2_ emissions. To further complicate the picture, biochar addition decreased N_2_ emissions from the Ferralsol (Fig. 5, Fig. 6).

## 4 Discussion

### 4.1 Biochar effects on N_2_O emission are largely decoupled from biochar effects on soil inorganic N availability and gross turnover

One important finding of our study was that biochar had a consistent mitigation effect on N_2_O emission (ca 70% reduction) independent of the soil type and amount of biochar (Fig. 1). It is remarkable that this was observed for all three soils given their different initial N_2_O emissions, properties and management history. This is generally consistent with earlier studies reporting that biochar reduced net N_2_O emissions at the soil-atmosphere interface, although the mitigation of N_2_O emissions in this study was higher than the average effect reported (Case et al., 2012; Saarnio et al., 2013; Cayuela et al., 2013; Cayuela et al., 2015; Hagemann et al., 2017). Some of the proposed mechanisms underlying N_2_O emission reduction include the reduction of mineral N (NH_4_^+^ and NO_3_^−^) availability, thus reducing the availability of N substrates for nitrification and denitrification (Singh et al., 2010). This mechanism relates on the one hand to biochar/soil surface/colloidal chemistry (e.g. pH and redox potential). On the other hand, through addition of C, also heterotrophic microbial immobilization could increase after biochar addition, thereby also reducing soil mineral N availability. Furthermore, the different redox-active components of biochar directly affect denitrification and its single steps – e.g., through a promotion of nitrate and N_2_O reduction via electron donation, a decrease in total denitrification by serving as alternative electron acceptor, or – most universally – by acting as electron shuttle for the nosZ harbouring bacterial community, thereby increasing gross N_2_O reduction and net N_2_O exchange at the soil-atmosphere interface (Cayuela et al., 2013; Chen et al., 2017). The latter universal process might dominate in our study in view of the consistent N_2_O reduction across soils, while biochar effects on soil mineral N availability were inconsistent and multidirectional (Fig. 5). Further or associated mechanisms how biochar impacts N_2_O reduction in denitrification have been reported and encompass e.g., entrapment in water-saturated soil pores and consequent stimulation of microbial N_2_O reduction by classical denitrifiers and atypical N_2_O reducers (Harter et al. 2016).

The second important observation of our study is that N_2_O emission was not directly coupled to dynamics gross microbial N turnover (ammonification, nitrification and microbial N immobilization). This might reflect that denitrification dominates N_2_O emissions with denitrification and in particular the N_2_:N_2_O ratios not directly depending on ammonification and nitrification. A decoupling of denitrification from ammonification and nitrification seems also possible in view of denitrification rates being several orders of magnitude lower than gross soil N turnover, and due to the different environmental and soil biogeochemical controls (Butterbach-Bahl et al., 2013).

For soil NH_4_^+^ concentrations, there was a persistent and significant trend for reduced concentrations under biochar addition across soils (Fig. 5). However, this did not affect gross nitrification as a potential source process for N_2_O, which was either increased (Acrisol10 soil) or overall unchanged (Acrisol100 and Ferralsol). The biochar-induced reduction of soil N_2_O emissions was also uncoupled from biochar effects on gross ammonification, which was either increased (Acrisol10and Ferralsol) or decreased (Acrisol100soil) by biochar (Fig. 5). Consequently, the persistent biochar-induced reduction of N_2_O emissions across three different agricultural soils, which had contrasting soil properties, gross N turnover and inorganic N availability, is supporting a rather universal mechanism that is acting during gross N_2_O formation and consumption through denitrification such as the “electron shuttle theory” (Cayuela et al., 2013). Sun et al. (2017) showed that biochars were able to rapidly transport electrons not only via surface functional groups but also through the carbon matrix, increasing electron transport in soils.

We have previously shown (Fungo et al., 2014) that steam-activation of biochar increases biochar's capacity to mitigate N_2_O emission. This suggests that the “*electron shuttle*” mechanism is facilitated by the surface chemistry of biochar to reduce activation energy required to cause cleavage of the N_2_O molecule to form N_2_. In fact, Chen et al. (2017) have shown that redox-active components (dissolved aromatic moieties and condensed aromatic structure) decreased total N denitrified because their dominant quinone moieties and electrical conductivity structure served as alternative electron acceptors. Chen et al. (2017) further observed that the redox-active components of biochar accelerated the last step of denitrification and decreased N_2_O emission by 74%–99%. In all cases their study showed a significant increase in organic matter-oxidizing and nitrate-reducing bacteria in the nosZ-harbouring bacterial community, which promoted N_2_O reduction.

A promotion of N_2_O reduction to N_2_ by biochar should result in increased N_2_ emissions. The data on N_2_ emissions available in the current study, however, support this for only the Acrisol 10. This is attributed to the high CEC due to secondary minerals in the Ferralsol compared to the Acrisol. There is needs to note, however, that N_2_ emissions are usually at least an order of magnitude larger than net N_2_O exchange at the soil-atmosphere interface (Fig. 5). This means that a small increase in gross N_2_O consumption due to biochar addition might hardly change the larger N_2_ emissions in this study (see also Wen et al., 2016). Although the spatiotemporal resolution of our N_2_ data preclude firm conclusions, the observed patterns tend to support that – independently of biochar effects on N_2_O reduction – there might be further effects of biochar on total denitrification, which again seems to be variable across the soils under investigation.

### 4.2 Biochar effects on gross N turnover

Though biochar effects on gross N turnover were variable across soils and biochar addition rate, we observed a remarkably strong stimulation of gross ammonification by a factor of 3–6 induced by biochar addition in the Ferralsol and a stimulation of gross nitrification in the Acrisol10 soil by 70% at least under 4% addition. Soil physicochemical properties may affect gross N turnover and availability of N via interaction with the minerals (Kizito et al., 2014), physical entrapment of substrates, diffusion in micro-pores (Fidel et al., 2017), and availability of easily mineralizable organic carbon (Lan et al., 2017). Increased nitrification and ammonification following biochar amendment has also been reported in previous studies. The suggested mechanisms include (i) provision of energy for microorganisms to degrade existing SOM through co-metabolism (Clough and Condron, 2010; Anderson et al., 2011; Nelissen et al., 2012); and (ii) absorbing potential allelochemical inhibitors of microbial metabolic pathways, such as monoterpenes and various polyphenolic compounds that are inhibiting nitrification (Ball et al., 2011).

A stimulation of microorganisms might also be based on the micronutrients such as Ca, Mg, Cu and B that are supplied by biochar. In the case of the Ferralsol, with the high clay content, CEC due to dominance of kaolinite and sesquioxides, low C and N contents and low inorganic N availability, the absorption capacity of clay minerals for available OC and NH_4_^+^ might explain the very low gross N turnover rates in the 0% biochar control treatment compared to the other two soils. Consequently, biochar addition indeed might have stimulated the microbial community by addition of C substrates, as all heterotrophic processes (ammonification, immobilization, denitrification) responded positively to the biochar treatment (Fig. 5).

## 5 Conclusions and recommendations

Our study demonstrates that biochar consistently reduced N_2_O emission in three different agricultural soils of western Kenya. As this effect was decoupled from biochar effects on gross soil N turnover and inorganic N concentrations, it may have been due to a universal mechanism such as the promotion of N_2_O reduction within the last step of denitrification, i.e., the “electron shuttle theory”. Biochar effects on gross N turnover were, in contrast to those on N_2_O emissions, very variable across soils. Despite a large number of analyzed soil parameters, it remained difficult to disentangle the mechanisms of these different biochar effects on gross N turnover, which makes it difficult to predict biochar effects on soil functions related to soil microbial inorganic N production and consumption.

## References

[bb0005] Ameloot N., De Neve S., Jegajeevagan K., Yildiz G., Buchan D., Funkuin Y.N., Prins W., Bouckaert L., Sleutel S. (2013). Short-term CO_2_ and N_2_O emissions and microbial properties of biochar amended sandy loam soils. Soil Biol. Biochem..

[bb0010] Anderson C.R., Condron L.M., Clough T.J., Fiers M., Steward A., Hill R.A., Sherlock R.R. (2011). Biochar induced soil microbial community change: implications for biogeochemical cycling of carbon, nitrogen and phosphorus. Pedobiologia.

[bb0015] Bagreev A., Bashkova S., Locke D.C., Bandosz T.J. (2001). Sewage sludge derived materials as efficient adsorbent for removal of hydrogen sulfide. Environ. Sci. Technol..

[bb0020] Ball P.N., MacKenzie M.D., DeLuca T.H. (2011). Wildfire and charcoal enhance nitrification and ammonium-oxidizing bacterial abundance in dry montane forest soils. J. Environ. Qual..

[bb9010] Brooks P.D., Star J.M., McInteer B.B., Preston T. (1989). Diffusion method to prepare soil extracts for automated nitrogen-15 analysis. Soil Sci. Soc. Am. J..

[bb0025] Butterbach-Bahl K., Dannenmann M. (2011). Denitrification and associated N_2_O emissions from agricultural sources in a changing climate. Curr. Opin. Environ. Sustain..

[bb0030] Butterbach-Bahl K., Willibald G., Papen H. (2002). Soil core method for direct simultaneous determination of N_2_ and N_2_O emissions from forest soils. Plant Soil.

[bb0035] Butterbach-Bahl K., Baggs E.M., Dannenmann M., Kiese R., Zechmeister-Boltenstern S. (2013). Nitrous oxide emissions from soils, how well do we understand the processes and their controls. Philos. Trans. R. Soc., B.

[bb0040] Case S.D.C., McNamara N.P., Reay D.S., Whitaker J. (2012). The effect of biochar addition on N_2_O and CO_2_ emissions from a sandy loam soil – the role of soil aeration. Soil Biol. Biochem..

[bb0045] Case S.D.C., McNamara N.P., Reay D.S., Stott A.W., Grant H.K., Whitaker J. (2015). Biochar suppresses N_2_O emissions while maintaining N availability in a sandy loam soil. Soil Biol. Biochem..

[bb0050] Castaldi S., Riondino M., Baronti S., Esposito F.R., Marzaioli R., Rutigliano F. a, Vaccari F.P., Miglietta F. (2011). Impact of biochar application to a Mediterranean wheat crop on soil microbial activity and greenhouse gas fluxes. Chemosphere.

[bb0055] Cayuela M.L., Sánchez-Monedero M.A., Roig A., Hanley K., Enders A., Lehmann J. (2013). Biochar and denitrification in soils: when, how much and why does biochar reduce N_2_O emissions?. Sci. Rep..

[bb0060] Cayuela M.L., Jeffery S., van Zwieten L. (2015). The molar H:C_org_ ratio of biochar is a key factor in mitigating N_2_O emissions from soil. Agric. Ecosyst. Environ..

[bb0065] Chen G., Zhang Z., Zhang Z., Zhang R. (2017). Redox-active reactions in denitrification provided by biochars pyrolyzed at different temperatures. Sci. Total Environ..

[bb0070] Ciais P., Sabine G. C., Bopp Bala L., Brovkin V., Canadell J., Chhabra A., DeFries R., Galloway J., Heimann M., Jones C., Le Quéré C., Myneni R.B., Piao S., Thornton P., Stocker T.F., Qin D., Plattner G.-K., Tignor M., Allen S.K., Boschung J., Nauels A., Xia Y., Bex V., Midgley P.M. (2013). Carbon and other biogeochemical cycles. Climate Change 2013: The Physical Science Basis. Contribution of Working Group I to the Fifth Assessment Report of the Intergovernmental Panel on Climate Change.

[bb0075] Clough T.J., Condron L.M. (2010). Biochar and the nitrogen cycle: introduction. J. Environ. Qual..

[bb0080] Cornelissen G., Rutherford D.W., Arp H.P.H., Dörsch P., Kelly C.N., Rostad C.E. (2013). Sorption of pure N_2_O to biochars and other organic and inorganic materials under anhydrous conditions. Environ. Sci. Technol..

[bb0085] Dannenmann M., Gasche R., Ledebuhr A., Papen H. (2006). Effects of forest management on soil N cycling in beech forests stocking on calcareous soils. Plant Soil.

[bb0090] Dannenmann M., Butterbach-Bahl K., Gasche R., Willibald G., Papen H. (2008). Dinitrogen emissions and the N_2_: N_2_O emission ratio of a Rendzic Leptosol as influenced by pH and forest thinning. Soil Biol. Biochem..

[bb9005] Dannenmann M., Willibald G., Sippel S., Butterbach-Bahl K. (2010). Nitrogen dynamics at undisturbed and burned Mediterranean shrublands of Salento Peninsula, Southern. Plant Soil.

[bb0095] Dannenmann M., Willibald G., Sippel S., Butterbach-Bahl K. (2011). Nitrogen dynamics at undisturbed and burned Mediterranean shrublands of Salento peninsula, southern Italy. Plant Soil.

[bb0100] Davidson E.A. (1992). Sources of nitric-oxide and nitrous-oxide following wetting ofdry soil. Soil Sci. Soc. Am. J..

[bb0105] Fidel R.B., Laird D.A., Thompson M.L., Lawrinenko M. (2017). Chemosphere Characterization and Quantification of Biochar Alkalinity.

[bb0110] Fungo B., Guerena D., Thiongo M., Lehmann J., Neufeldt H., Kalbitz K. (2014). N_2_Oand CH_4_ emission from soil amended with steam-activated biochar. J. Plant Nutr. Soil Sci..

[bb0115] Guo C.J., Dannenmann M., Gasche R., Zeller B., Papen H., Polle A., Rennenberg H., Simon J. (2013). Preferential use of root litter compared to leaf litter by beech seedlings and soil microorganisms. Plant Soil.

[bb0120] Hagemann N., Harter J., Kaldamukova R., Guzman-bustamante I., Ruser R., Graeff S. (2017). Does Soil Aging Affect the N 2 O Mitigation Potential of Biochar? A Combined Microcosm and Field Study.

[bb0125] Harter J., Krause H.M., Schuettler S., Ruser R., Fromme M., Scholten T. (2014). Linking N_2_O emissions from biochar-amended soil to the structure and function of the N-cycling microbial community. Int. Soc. Microb. Soc. J..

[bb0130] Harter J., Krause H.M., Schuettler S., Ruser R., Fromme M., Scholten T., Kappler A., Behrens S. (2014). Linking N_2_O emissions from biochar-amended soil to the structure and function of the N-cycling microbial community. ISME J..

[bb0135] Harter J., Guzman-Bustamante I., Kuehfuss S., Ruser R., Well R., Spott O., Kappler A., Behrens S. (2016). Gas entrapment and microbial N_2_O reduction reduce N_2_O emissions from a biochar-amended sandy clay loam soil. Sci. Rep..

[bb0145] Hitoshi T., Ai F., Haruo H. (2002). Development of advanced utilization technologies for organic waste: (part I) greenhouse gas and nutrient salt adsorption properties of wood-based charcoal. Denryoku Chuo KenkyujoHokoku, Research Report of Abiko Research Laboratory No. 0201.

[bb0150] Hoffmann G. (1991). *Methodenbuch Band 1, Die Untersuchung von Böden.*Auflage.

[bb0155] Karhu K., Mattila T., Bergström I., Regina K. (2011). Biochar addition to agricultural soil increased CH_4_ uptake and water holding capacity e results from a short- term pilot field study. Agric. Ecosyst. Environ..

[bb0160] Kirkham D., Bartholomew W.V. (1954). Equations for following nutrient transformations in soil utilizing tracer data. Soil Sci. Soc. Am. Proc..

[bb0165] Kizito S., Wu S., Kipkemoi K.,.W., Lei M., Lu Q., Bah H., Dong R. (2014). Evaluation of slow pyrolyzed wood and rice husks biochar for adsorption of ammonium nitrogen from piggery manure anaerobic digestate slurry. Sci. Total Environ..

[bb0170] Lan Z.M., Chen C.R., Rashti M.R., Yang H., Zhang D.K. (2017). Science of the Total environment stoichiometric ratio of dissolved organic carbon to nitrate regulates nitrous oxide emission from the biochar-amended soils. Sci. Total Environ..

[bb0175] Lehmann J., Rillig M.C., Thies J., Masiello C.a., Hockaday W.C., Crowley D. (2011). Biochar effects on soil biota – a review. Soil Biol. Biochem..

[bb0180] Nelissen V., Rütting T., Huygens D., Staelens J., Ruysschaert G., Boeckx P. (2012). Maize biochars accelerate short-term soil nitrogen dynamics in a loamy sand soil. Soil Biol. Biochem..

[bb0190] Saarnio S., Heimonen K., Kettunen R. (2013). Biochar addition indirectly affects N_2_O emissions via soil moisture and plant N uptake. Soil Biol. Biochem..

[bb0195] Singh B.P., Hatton B.J., Singh B., Cowie A.L., Kathuria A. (2010). Influence of biochar on nitrous oxide emission and nitrogen leaching from two contrasting soils. J. Environ. Qual..

[bb0200] Sun T., Levin B.D.A., Guzman J.J.L., Enders A., Muller D.A., Angenent L.T., Lehmann J. (2017). Rapid electron transfer by the carbon matrix in natural pyrogenic carbon. Nat. Commun..

[bb0205] Van Zwieten L., Singh B., Joseph S., Kimber S., Cowie A., Chan K.Y., Lehmann J., Joseph S. (2009). Biochar and emissions of non-CO_2_ greenhouse gases from soil. Biochar for Environmental Management: Science and Technology.

[bb0210] Wen Y., Chen Z., Dannenmann M., Carminati A., Willibald G., Kiese R., Wolf B., Veldkamp E., Butterbach-Bahl K., Corre M. (2016). Disentangling gross N_2_O production and consumption in soil. Sci. Rep..

[bb0215] Wu F., Jia Z., Wang S., Chang S.X., Startsev A. (2012). Contrasting effects of wheat straw and its biochar on greenhouse gas emissions and enzyme activities in a Chernozemic soil. Biol. Fertil. Soils.

[bb0225] Yanai Y., Toyota K., Okazaki M. (2007). Effects of charcoal addition on N_2_O emissions from soil resulting from rewetting air-dried soil in short-term laboratory experiments. Soil Sci. Plant Nutr..

[bb0230] Yao Z.S., Wu X., Wolf B., Dannenmann M., Butterbach-Bahl K., Brüggemann N., Chen W., Zheng X. (2010). Soil-atmosphere exchange potential of NO and N_2_O in different land use types of Inner Mongolia, as affected by soil temperature, soil moisture, freeze-thaw and drying-rewetting events. J. Geophys. Res.-Atmos..

[bb9000] Yu X.-Y., Mu C.-L., Gu C., Liu C., Liu X-J. (2011). Impact of woodchip biochar amendment on the sorption and dissipation of pesticide acetamiprid in agricultural soils. Chemosphere.

